# The phylogenetic relationship among two species of genus *Nebo* (Scorpiones: Diplocentridae) from Saudi Arabia and Middle East

**DOI:** 10.1186/s40850-023-00166-9

**Published:** 2023-04-07

**Authors:** Abdulaziz R. Alqahtani, Noura J. Alotaibi, Hamdy Aly, Ahmed Badry

**Affiliations:** 1 Department of Biology, College of Science, University of Bisha, P.O. Box 551, Bisha, 61922 Saudi Arabia; 2 Department of Biology, Faculty of Science, Taif University, P.O. Box 11099, Taif, 21944 Saudi Arabia; 3 Department of Zoology, Faculty of Science, Al-Azhar University, Assiut, Egypt; 4 Department of Zoology, Faculty of Science, Al-Azhar University, Nasr City, Cairo, Egypt

**Keywords:** Scorpiones, Diplocentridae, Diplocentrinae, Nebinae, *Nebo*, Phylogenetic, mtDNA

## Abstract

**Background:**

The genus *Nebo* has been identified as a medically important scorpion species distributed across Arabia and the Middle East. However, its taxonomic status remains unclear.

**Aim:**

The molecular phylogeny of two *Nebo* species from Saudi Arabia and comparative sequences from Palestine is presented based on the mitochondrial cytochrome oxidase subunit I (COI) gene.

**Methodology:**

Scorpion specimens were collected from two different localities, mainly the Southern part of Saudi Arabia. Then, DNA was extracted, amplified using invertebrate universal primers, and sequenced to identify the COI gene. The obtained sequences were analyzed, and phylogenetic trees based on maximum parsimony, neighbor-joining, and Bayesian inference were constructed.

**Results:**

The inferred phylogeny indicates the monophyletic status of the family Diplocentridae and its subfamily Nebinae and Diplocentrinae. Also, the phylogenetic analyses support the existence of interspecific and intraspecific variations among/ within *Nebo hierichonticus* and *Nebo yemenensis* which may indicate distinct species.

**Conclusion:**

Further morphological studies with additional specimens from the Arabian Peninsula may reveal possible undiscovered and cryptic species.

## Introduction

Arthropod is one of the diverse group on earth [1, 2] and Arachnida is one of the important class belonging to it. With more than 2,700 species, scorpions are a category of arachnids that have significant evolutionary success within invertebrates [3]. With about 138 species in 10 genera, Diplocentridae Karsch, 1880 is considered native to the New World and Middle East [4]. After revising higher scorpion systematics, Soleglad & Fet [5] abolished the family Diplocentridae and merged all genera into the Scorpionidae family. Despite being closely related to Scorpionidae, Diplocentridae differ from them because they have subaculear tubercles on the telsons [6]. Many researchers are considering Diplocentridaee as a valid family [7, 8].

There are several species belonging family Diplocentridae that have been identified and reported as medically significant scorpion species [9]. The genus *Nebo* contains nine species distributed across Arabia and the Middle East [10–14]. This genus has been difficult to identify taxonomically because of the limited number of specimens available, morphometric ratios used and other morphological characters such as carination, trichobothrial patterns, pectinal tooth counts, and tarsomere II spine formulas [15–17]. Kinzelbach [18] and Vachon & Kinzelbach [19] proposed to treat all taxa belong genus *Nebo* from full species status to subspecies of *N*. *hierichonticus* (Simon, 1872). However, Francke [15] and Sissom [16] considered allopatric populations to have consistent morphological characters, morphometric ratios and thus considered them to be valid species. In Saudi Arabia, two species of *Nebo* have been reported; *N. hierichonticus* and *N. yemenensis* [15, 20, 21]. Based on the specimens with very limited sample size studied by [17], some of their diagnostic characteristics were closer to *N. hierichonticus* while others closer to *N. yemenensis*.

In addition, Arachnid orders like scorpions are understudied in comparison to other arachnid families, especially when it comes to their systematics and taxonomy [22]. Many scorpion taxa are characterized by high levels of morphological uniformity and conservatism, which hampers straightforward species delimitation [23]. More insights were gained later from studies of mitochondrial and nuclear gene variation [24]. Recent research has employed the molecular phylogeny to interpret the evolutionary relationships of numerous populations of scorpions [25–28];. Based on morphological studies *Nebo yemenensis* reported for first time from the Southwestern highlands of Asir and Jizan Provinces, Saudi Arabia by [21]. To evaluate the taxonomical status and the occurrence of *N. yemenensis*, with additional comparative sequence data of *N. hierichonticus* from Palestine retrieved from the Genbank.

## Material and methods

### Biological material

**Fig. 1:**
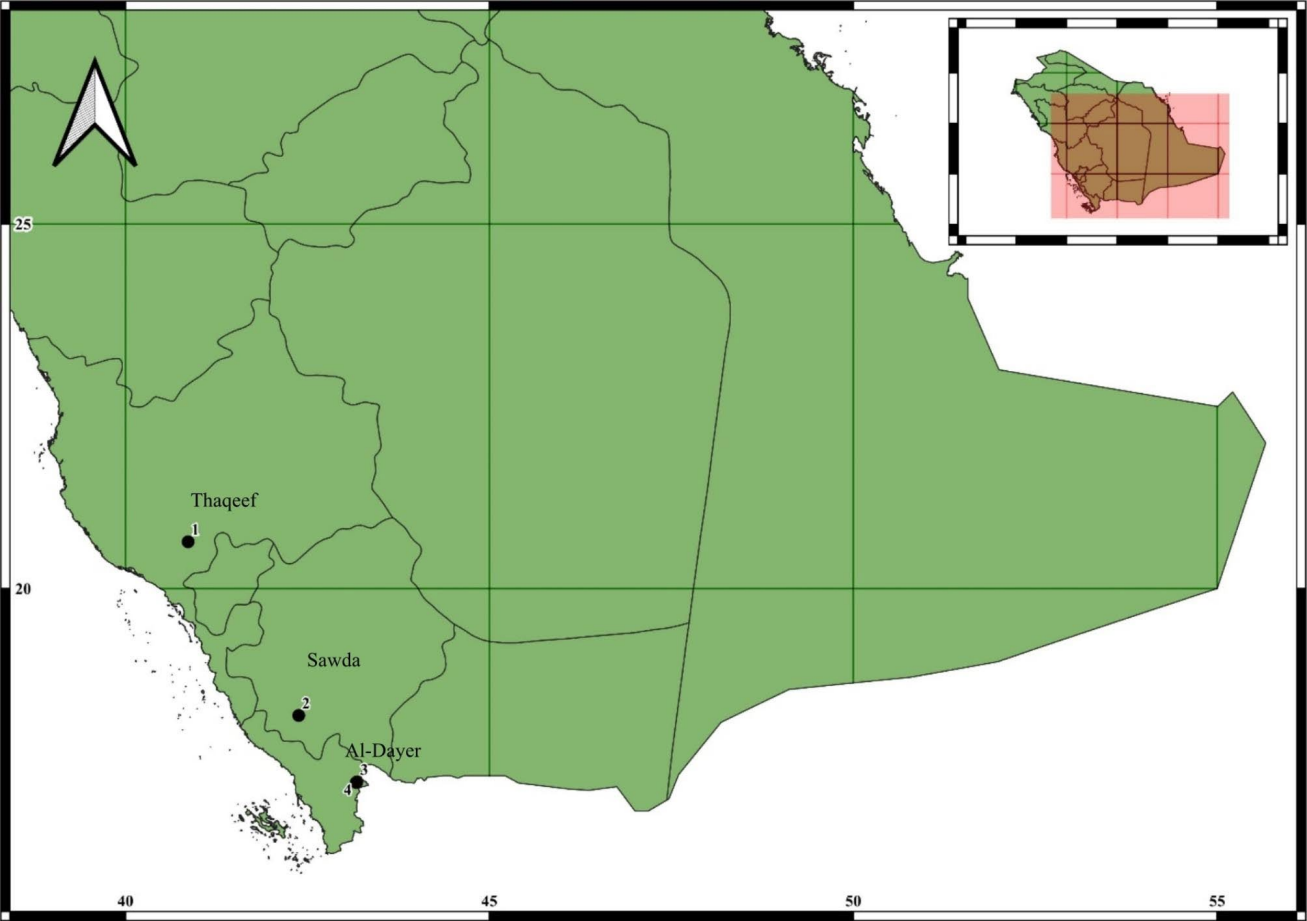
Collection localities of *Nebo yemenensis* samples collected from Saudi Arabia that are given in Table 1

Four adult specimens belonging to *N. yemenenesis* scorpion species were collected from two different regions in Saudi Arabia (Fig. 1; Table 1). The scorpions were collected mainly at night using ultraviolet from September 2021 to July 2022 and preserved according to the methods described [14]. The climate and habitat of this species were summarized and described by [21].

**Table 1:** Localities and GenBank accession numbers for *Nebo* samples collected from Saudi Arabia used in this study

Species	Site	Region	Latitude	Longitude	No. of Samples	Accession Number
*N. yemenensis*	Thaqeef	Mecca	20.642	40.86	1	OP970165
*N. yemenensis 1*	Sawda	Asir	18.26	42.38	1	OP970166
*N. yemenensis2*	Al-Dayer	Jizan	17.35	43.18	1	OP970167
*N. yemenensis 3*	Al-Dayer	Jizan	17.34	43.16	1	OP970168

### DNA extraction, amplification, and sequencing

The whole genomic DNA was extracted from freshly preserved (96% ethanol) scorpion specimens using Qiagen extraction kit (Qiagen) according to its manufacturer’s instructions. A fragment of COI gene was amplified via standard polymerase chain reaction (PCR) using invertebrate universal primers (LCO1490 and HCO2198) as determined by [29]. The amplified products of COI gene were checked, purified, and sequenced on an ABI 3500 automated sequencer (Applied Biosystems Inc., USA). The obtained sequences were deposited in GenBank (Table 1) at https://www.ncbi.nlm.nih.gov/genbank/ with accession numbers (OP970165, OP970166, OP970167, OP970168).

### Phylogenetic analysis

The obtained Sequences were screened and analyzed by eye using Finch TV 1. 4. 0 (Geospiza, Inc., USA; http://www.geospiza.com). Also, additional comparative sequences for the subfamily Nebianae represented by *Nebo hierichonticus* from Palestine (MT418015.1) and Diplocentridae were retrieved from GenBank as an ingroup. Also, *Scorpio palmatus* was downloaded as outgroup (KT188367.1). The sequence data was aligned using the default settings of ClustalW [30]. The nucleotide composition was calculated based on only the sequences within each ingroup. The pairwise genetic distances (p-distances) were calculated for the whole data set using Mega 6 [30]. All phylogenetic analyses were performed using three different methods based on the COI data set (n = 19), including maximum-parsimony, neighbor-joining, and Bayesian inference as described by [26].

## Results

### Genetic data

A total of 590 aligned nucleotides from the COI data set were analyzed. There were 379 (64.23%) constant bases, 211 (35.67%) variable bases, and 164 (27.79%) parsimonious bases. The composition of nucleotides was highly biased towards A–T bases. Among the sequence data, T, C, A, and G had mean values of 44.3, 13.3, 21.4, and 21.0%, respectively. It was found that 47 polymorphic segregating sites were detected in the 590 bp region. The genetic distance between *N*. *yemenensis* and the others of genus *Nebo* ranged from 4.0 to 6.0%. while the divergences among other diplocentrid taxa ranged from 0.12 to 0.17 (Table 2).

**Table 2:** The uncorrected p distance of the sequence divergence of COI mtDNA sequences between *Nebo* samples and other related Diplocentridae species included in this study

Species	1	2	3	4	5	6	7	8	9	10	11	12	13	14	15	16	17	18	19
***OP970166 N. yemenensis***		0.01	0.00	0.01	0.01	0.01	0.02	0.01	0.01	0.01	0.02	0.02	0.02	0.01	0.01	0.01	0.01	0.01	0.02
***OP970165 N.*** **yemenensis**	0.03		0.01	0.01	0.01	0.01	0.02	0.02	0.01	0.01	0.01	0.02	0.01	0.02	0.02	0.01	0.01	0.01	0.02
***OP970167 N. yemenensis***	0.01	0.04		0.01	0.01	0.01	0.02	0.02	0.01	0.01	0.02	0.02	0.02	0.01	0.02	0.01	0.01	0.01	0.02
***OP970168 N. yemenensis***	0.02	0.05	0.02		0.01	0.01	0.02	0.02	0.01	0.01	0.02	0.02	0.02	0.01	0.02	0.02	0.01	0.01	0.02
**MT418015.1** ***N. hierichonticus***	0.04	0.04	0.05	0.06		0.00	0.02	0.01	0.01	0.01	0.01	0.02	0.01	0.01	0.01	0.01	0.01	0.01	0.02
**AY156571.1** ***N. hierichonticus***	0.04	0.04	0.05	0.06	0.01		0.02	0.01	0.01	0.01	0.01	0.02	0.01	0.01	0.01	0.01	0.01	0.01	0.02
**KM514646.1** ***D. hoffmanni***	0.17	0.16	0.17	0.18	0.15	0.16		0.01	0.01	0.01	0.01	0.01	0.01	0.02	0.01	0.01	0.01	0.01	0.02
**KM514637.1** ***D. anophthalmus***	0.16	0.16	0.17	0.17	0.15	0.15	0.12		0.01	0.01	0.01	0.01	0.01	0.01	0.01	0.01	0.02	0.01	0.01
**KM514636.1** ***K. poncei***	0.12	0.13	0.13	0.14	0.12	0.12	0.13	0.10		0.01	0.01	0.01	0.01	0.01	0.01	0.01	0.01	0.01	0.01
**KM514635.1** ***K. magnus***	0.14	0.15	0.15	0.15	0.13	0.13	0.12	0.11	0.07		0.01	0.01	0.01	0.01	0.01	0.01	0.01	0.01	0.01
**KM514656.1** ***D. rectimanus***	0.15	0.15	0.16	0.17	0.14	0.14	0.06	0.11	0.12	0.11		0.01	0.01	0.01	0.01	0.01	0.02	0.01	0.01
**KM514641.1** ***D. coylei***	0.15	0.15	0.16	0.17	0.15	0.15	0.12	0.10	0.11	0.13	0.11		0.01	0.01	0.01	0.01	0.01	0.01	0.02
**KM514638.1** ***D.*** **bereai**	0.14	0.14	0.15	0.16	0.13	0.14	0.12	0.11	0.11	0.12	0.11	0.10		0.01	0.01	0.01	0.01	0.01	0.02
**KM514631.1** ***B. caboensis***	0.15	0.15	0.15	0.16	0.15	0.15	0.16	0.13	0.13	0.14	0.13	0.13	0.14		0.01	0.01	0.02	0.01	0.02
**KM514644.1** ***D.*** **formosus**	0.16	0.16	0.17	0.17	0.15	0.15	0.13	0.10	0.12	0.13	0.12	0.10	0.10	0.13		0.01	0.01	0.01	0.02
**KM514643.1** ***D. diablo***	0.15	0.15	0.16	0.17	0.14	0.14	0.11	0.10	0.12	0.11	0.10	0.12	0.10	0.14	0.11		0.01	0.01	0.02
**KM514629.1** ***H. jamaicae***	0.14	0.15	0.15	0.16	0.14	0.15	0.15	0.15	0.13	0.14	0.15	0.13	0.13	0.15	0.15	0.13		0.01	0.02
**KM514634.1*D***. ***lesueurii***	0.13	0.14	0.14	0.15	0.13	0.13	0.13	0.14	0.11	0.12	0.12	0.14	0.12	0.14	0.13	0.13	0.13		0.02
**KT188367.1*S***. ***palmatus***	0.16	0.16	0.17	0.18	0.16	0.16	0.17	0.16	0.15	0.15	0.16	0.17	0.17	0.18	0.18	0.18	0.17	0.15	

### Phylogenetic analyses

**Fig. 2:**
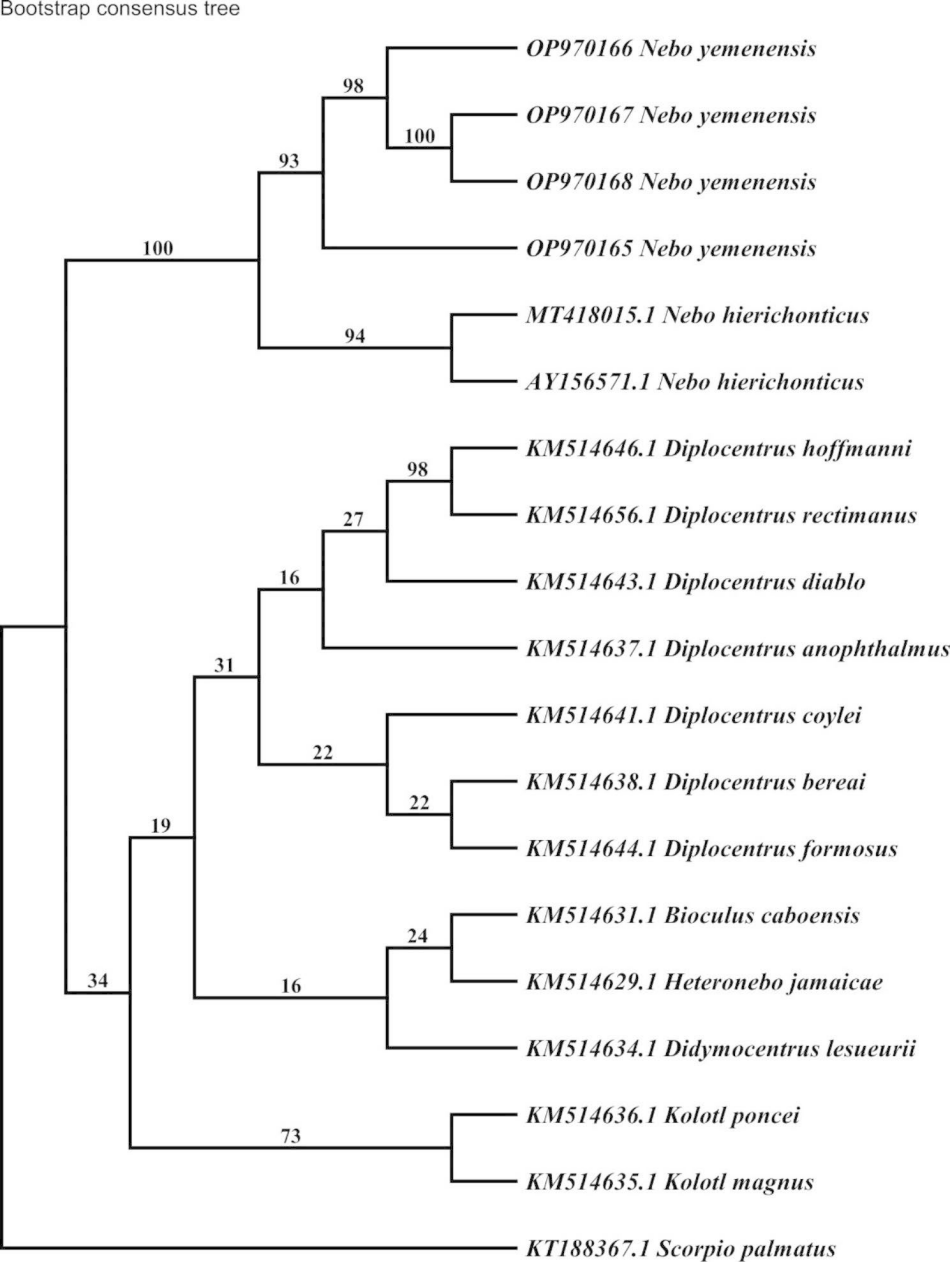
Maximum-parsimony phylogenetic tree of genus *Nebo* and other Diplocentrids related sequences of the COI gene from Saudi Arabia and Palestine. Number above branches indicate bootstrap values calculated with 1000 replicates

**Fig. 3:**
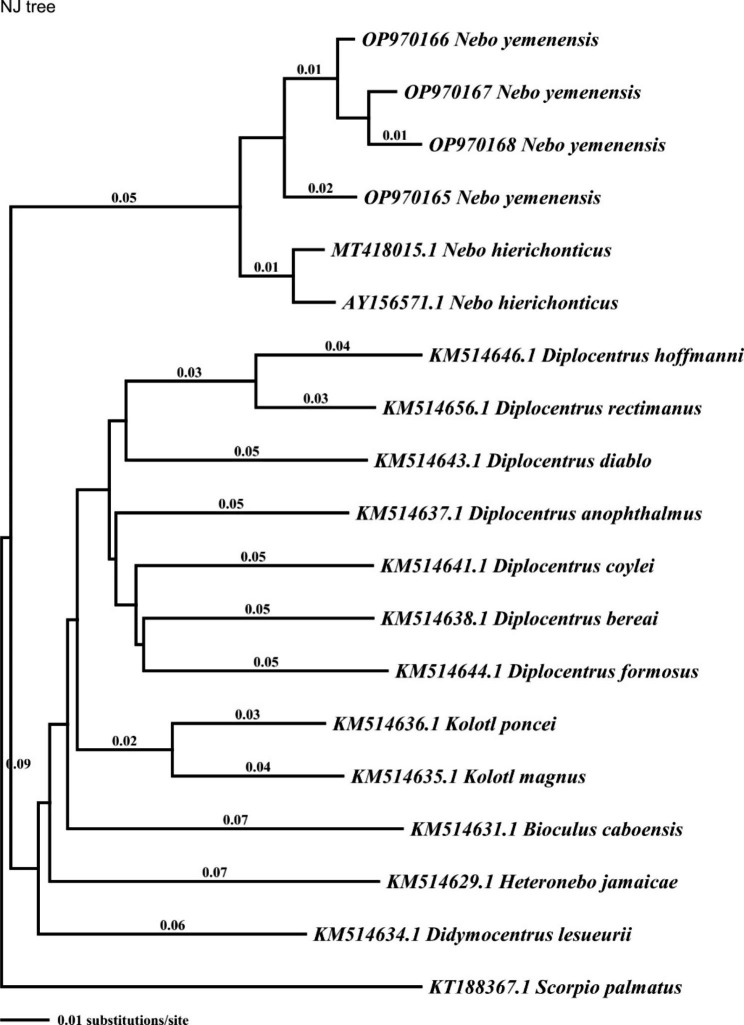
Neighbor-joining phylogenetic tree of genus *Nebo* and other Diplocentrids related sequences of the COI gene from Saudi Arabia and Palestine. Number above branches indicate distance values

**Fig. 4:**
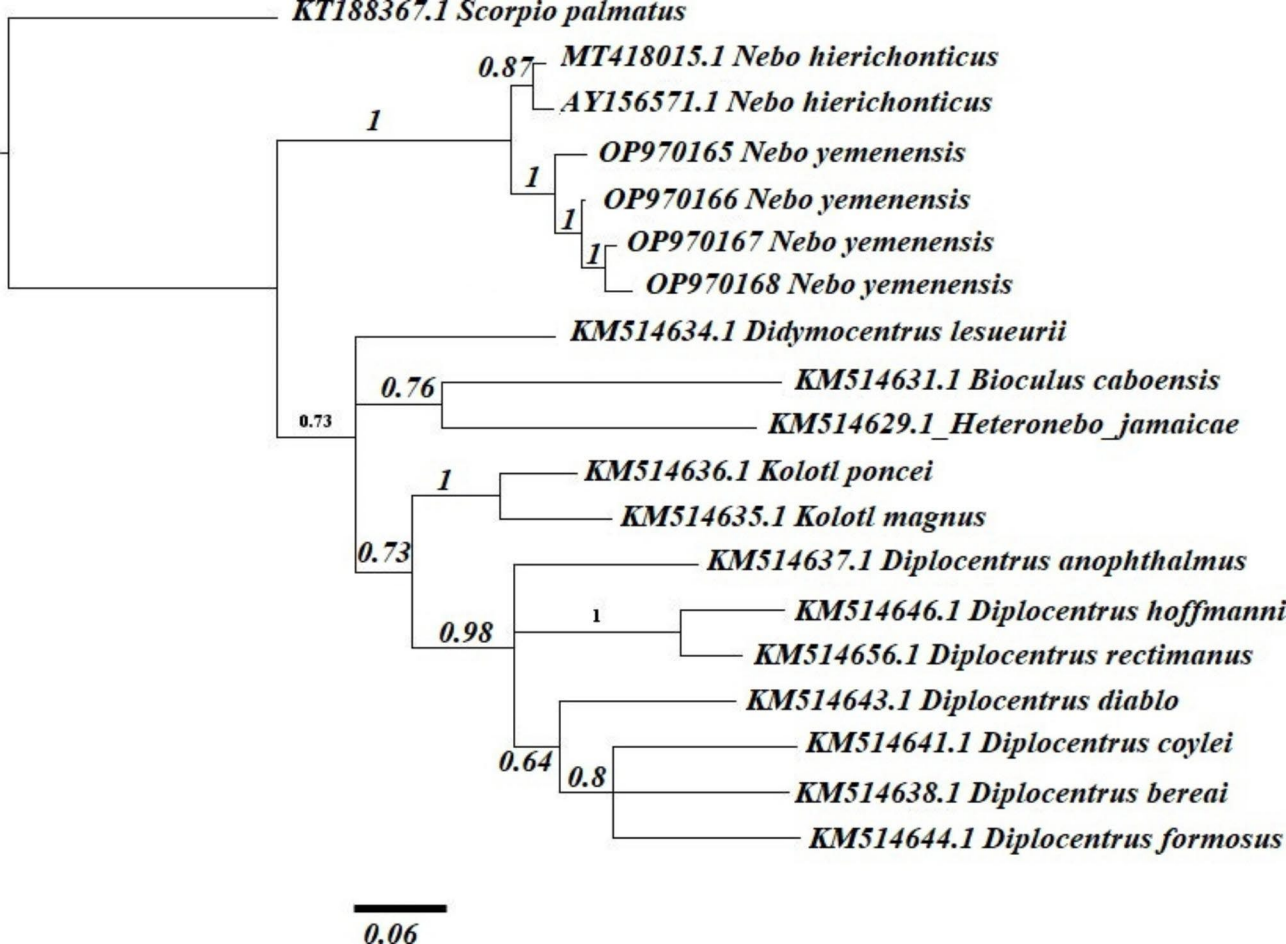
Bayesian inference phylogenetic tree of genus *Nebo* and other Diplocentrids related sequences of the COI gene from Saudi Arabia and Palestine Numbers above nodes indicate the posterior probabilities

We performed the maximum parsimony analysis within 590 bp in the length of the sequences, the gaps treated as missing which produced two most-parsimonious trees with a length of 625 steps (homoplasy index = 0.5152; consistency index = 0.4848; retention index = 0.5589). The resulting tree showed two major supported clades which are represented by Diplocentridae related taxa (Fig. 2). A first clade includes all taxa belonging to the subfamily Nobinae. This clade is split into two subclades, including *N. hierichonticus* from Palestine (MT418015.1) as a basal clade to *N*. *yemenensis* from Saudi Arabia. The later also divided into two subclades, including *N. yemenensis* sample from Thaqeef, Mecca (OP970165), as a basal clade the samples from Southwestern Saudi Arabia (OP970166- OP970168) which grouped as a sister group. While the second clade encompassed all taxa belonging to the family Diplocentrinae. Similarly, the neighbor-joining analysis generated a tree which showed the results of the neighbor-joining analysis (Fig. 3). The resulting tree has a general topology nearly identical to the maximum parsimony tree as shown in Fig. 2. The general topology of the Bayesian inference tree shown in Fig. 4 is very similar to both the maximum parsimony (Fig. 2) and neighbor-joining trees (Fig. 3). This analysis clearly showed that the clustering of subfamily Nebinae which represented samples belong genus *Nebo* from Palestine and Saudi Arabia, and the diplocentrids related taxa. The first clade is supported by a posterior probability value 1. While the second clade is weakly supported by posterior probability value 0.73.

## Discussion

The phylogenetic analyses based on maximum-parsimony, neighbor-joining, and Bayesian inference strongly to support the monophyly of family Diplocentridae based on COI gene as represented by different species of Diplocentridae taxa (Figs. 2, 3 and 4). Prendini [7] validated the monophyly of Diplocentridae due to three features: “a fused lamellar hook and a median lobe of the hemispermatophore, subaculear tubercle, and the red venom coloration”. Also, our analyses support the monophyly of the subfamily Nebinae and subfamily Diplocentrinae. It was also revealed that Nebinae is monophyletic since just one character (trichobotrium position *it* distal to *ib*) supports this hypothesis. However, internal relationships have not been resolved completely, revealing that some genera are paraphyletic. As a result of various diagnostic characteristics that split Nebinae and Diplocentrinae, Santibáñez-López et al. [8] concluded that the Nebinae subfamily is a synonym for Diplocentrinae, based on multilocus morphological and molecular phylogenetic analysis.

Within the Nebinae clade, *N. hierichonticus* was basal to the *N. yemenensis* from Saudi Arabia. *N*. *hierichonticus*, differs from the above in that it has diagnostically relevant morphometric ratios indicated by its holotype and paratype, except the length/width of the pedipalp chela is slightly higher than that determined by [15]. *N. hierichonticus*, a fossorial scorpion species found under self-dug deep caves in the deserts and arid to semiarid mountainous regions across Egypt (Sinai), Palestine, Jordan, Syria and Lebanon [31]. The genetic distance between *N*. *yemenensis* and the others of the genus *Nebo* ranged from 4.0 to 6.0% (Table 2). It has been reported previously that several studies on allied genera have yielded similar results (e.g. *Androctonus*, *Buthus*, *Buthacus Hottentotta, Leiurus*, and *Scorpio*), [26–28, 32–39].

Within the Saudi Arabian *N. yemenensis*, the sister group relationship is strongly supported between the samples collected from Mecca (OP970165) and Jizan (OP970166- OP970168). However, the divergence between *N*. *yemenensis* obtained from Mecca and those obtained Jizan, in Southwestern Saudi Arabia, ranged from 3 to 5%. The existence of cryptic species between these populations could explain this. Kinzelbach [18] have regarded several species in the genus and [19] as subspecies of *N. hierichonticus* (Simon, 1872). However, Francke [15] and Sissom [16] argued that these morphometric ratios were reliable and nonoverlapping among populations and therefore referred to them as valid species. The male hemispermatophore was also found to possess other useful characteristics consistent with Francke’s “morphometric” species. Francke [15] was unable to assign *Nebo* from Saudi Arabia to any taxon due to the very limited sample size (some characters resembled *N*. *hierichonticus*, while others resembled *N. yemenensis*). However, it was suggested that their eventual location and determination were significant because it reduced the large discontinuity between *N*. *hierichonticus* in the north and the other species in the south (Saudi Arabia, Yemen, and Oman). [39–41] referred to the existence of three distinct clusters of *Androctonus crassicauda* populations collected from different ecogeographical regions in Saudi Arabia based on molecular and morphological investigations. In other words, the variation between species in the genus *Nebo* may reflect vicariances and dispersals caused by climatic and geologic changes that have shaped the Arabian landscape during the past few million years.

## Conclusion

In conclusion, this study demonstrated a clear intraspecific and interspecific variation among two species of genus *Nebo* from Saudi Arabia and Palestine, with strong support for a monophyletic relationship of the subfamily Nebinae and Diplocentrinae pending additional analyses with more representative species. The relationships between Saudi *N*. *yemenensis* and other species of this genus need to be clarified, which will require additional in-depth morphological and molecular revisions.

## Data Availability

The dataset generated and analyzed during the current study in the https://www.ncbi.nlm.nih.gov/genbank/ with accession numbers (OP970165, OP970166, OP970167, OP970168).
